# Advanced stage cutaneous T-cell lymphoma treated with high-dose external beam radiation therapy: A case report

**DOI:** 10.1097/MD.0000000000032239

**Published:** 2022-12-23

**Authors:** Sang Hee Youn, Sun Young Lee

**Affiliations:** a Departments of Radiation Oncology, Jeonbuk National University Hospital-Jeonbuk National University Medical School, Jeonju, Republic of Korea; b Research Institute of Clinical Medicine of Jeonbuk National University-Biomedical Research Institute of Jeonbuk National University Hospital, Jeonju, Republic of Korea.

**Keywords:** case report, cutaneous T-cell lymphoma, external beam radiation therapy, high-dose, three-dimensional conformal radiotherapy

## Abstract

The treatment for advanced-stage or refractory cutaneous T-cell lymphoma (CTCL) is largely palliative. Due to the rarity of the disease, there are few randomized controlled trials and large-scale prospective studies. Therefore, the treatment of advanced-stage CTCL remains challenging.

**Patient concerns and diagnosis:**

A 64-years-old man who had received narrowband UVB phototherapy for several years presented with a generalized rash with widespread polycyclic erosions and painful ulcers on his hands and feet. We restaged him as stage III CTCL.

**Interventions and outcomes:**

He was treated with high-dose radiation for curative purposes and 3-dimensional conformal radiation therapy to both hands and feet. At the 2-year follow-up after the end of radiotherapy, the irradiated skin had recovered to normal soft, smooth skin with localized fibrosis and hypopigmented skin. He reported an excellent quality of life, and his hands and feet were free to move.

**Conclusion:**

CTCL at an advanced stage could require dose escalation with local radiotherapy for curative purposes. High-dose 3-dementional conformal radiation therapy could be effective and has tolerable toxicities.

## 1. Introduction

Cutaneous T-cell lymphoma (CTCL) is a heterogeneous group of neoplasms with monoclonal T-cell lymphoproliferation involving the skin.^[1]^ Primary CTCL present in the skin without extracutaneous disease at the time of diagnosis.^[2]^ The majority of CTCL patients are diagnosed at an early stage and have an excellent prognosis. The treatment for advanced-stage or refractory CTCL is largely palliative. Due to the rarity of the disease, there are few randomized controlled trials and large-scale prospective studies. Therefore, the treatment of advanced-stage CTCL remains challenging.

Technical modifications of local radiotherapy have allowed for the optimization of dose distribution, resulting in improved clinical outcome and reduction in the number of chronic complications. The intensity of radiation must be chosen to provide an adequate dose to the bottom of the lesion. An electron beam can be used successfully for the treatment of superficial and flat cutaneous lesions of CTCL; however, for deeper and irregular contoured lesions, electron beam irradiation provides an indistinct dose distribution and ineffective dose delivery to deeper lesions. However, a photon beam is usually used for the treatment of deep and irregular lesions. Additionally, the 3-dementional conformal radiation therapy (3D-CRT) protocol begins with individualized, 3D digital datasets, which are used in the generation of 3D computer images of the patient’s lesions and the normal adjacent tissue anatomy. By using 3D-CRT with photon beams, an adequate amount of therapeutic radiation can be delivered to lesions that are deeper and have irregular contours. CTCLs are radiosensitive, but the hands and feet are usually difficult to irradiate with conventional electron beam fields. In this case report, the patient was treated with high-dose radiation for curative aim,^[3]^ and 3D-CRT was also applied to both hands and feet for more effective local control of the lesions in the interdigital skin area than conventional electron therapy.^[4]^

We present an aggressive and refractory case of a 64-years-old man who had received narrowband UVB phototherapy for several years but who presented with a generalized rash with widespread polycyclic erosions and ulcers on his hands and feet when he first came to our center of radiation oncology. In this case report, we described remission in a patient with tumor-stage CTCL treated with high-dose 3D-CRT.

## 2. Case presentation

A 64-years-old man developed a pruritic generalized patch 10 years prior to presentation to the hospital. He was diagnosed with stage IB CTCL at the time of diagnosis, and there was no evidence of lymph node involvement on his physical examination and FDG-PET/CT imaging. He was treated with narrowband UVB phototherapy for 3 years. He is on medication for diabetes but had no other past medical history. And he had no family history of malignant disease within the second degree of kinship. He was an office worker with no smoking history.

**Figure 1.**
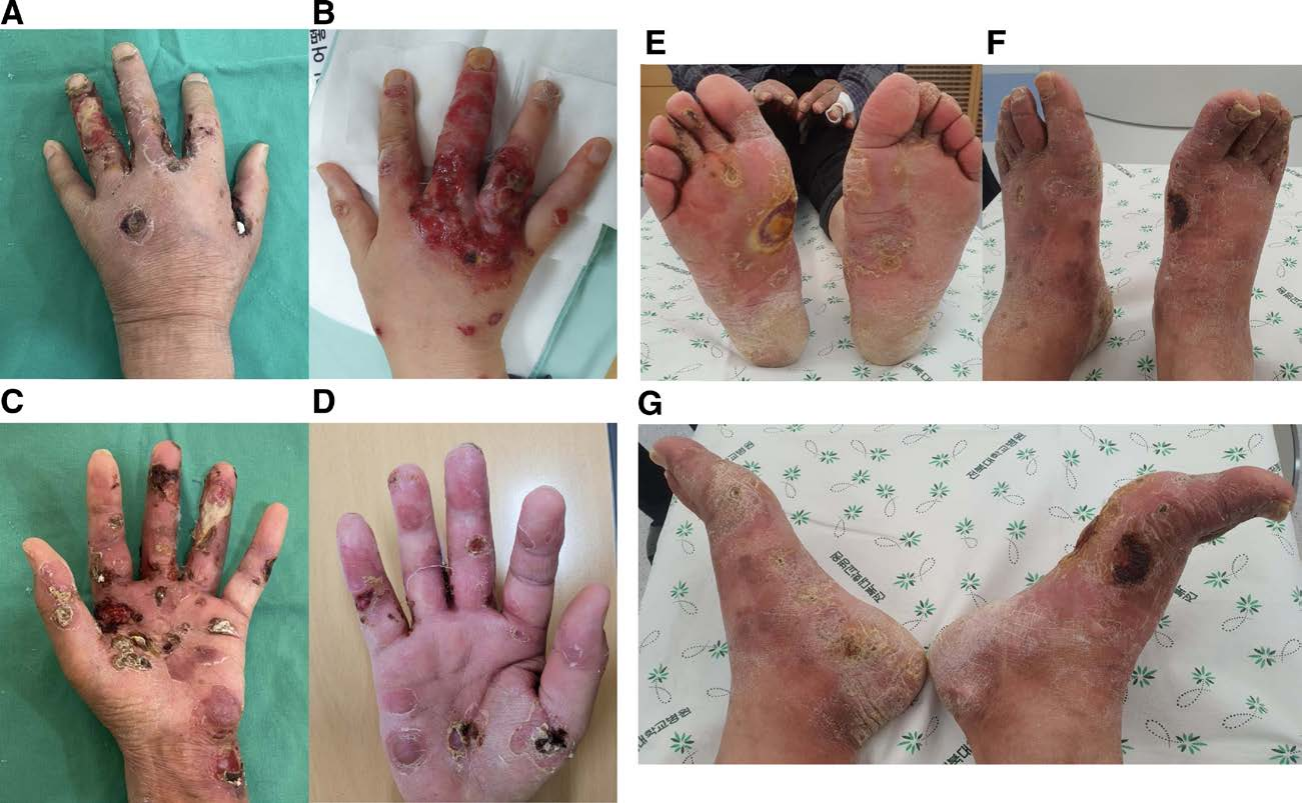
Clinical presentation of CTCL. Shown are the patient’s edematous hands and feet with polycyclic, well-defined erosion and ulcers. (A) The back of left hand; (B) the back of right hand; (C) left palm; (D) right palm; (E) both soles; (F) the top of both feet; (G) the inside of both feet. CTCL = cutaneous T-cell lymphoma.

**Figure 2.**
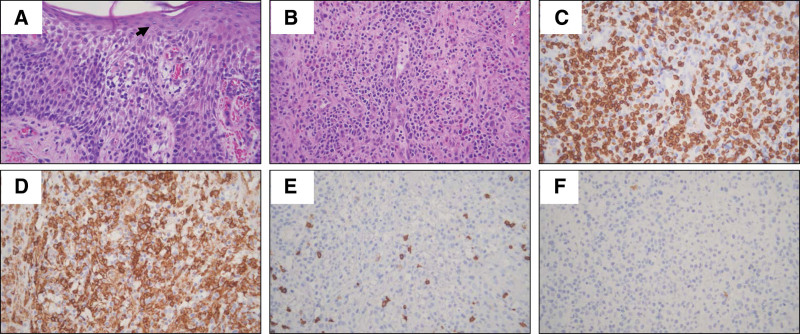
Histologic findings of cutaneous T-cell lymphoma. (A) Atypical lymphoid cells infiltrate the epidermis and form a Pautrier microabscess (arrow). (B) Atypical lymphoid cells infiltrate the dermis. (C–F) Immunohistochemically, tumor cells are positive for CD3 (C) and CD4 (D) but negative for CD8 (E) and CD20 (F). Original magnification, ×400.

Despite the use of phototherapy, the ulcers continued to develop and spread gradually on both hands and feet. Hemorrhage and pain in both feet caused him to have difficulty walking, and the edema and pain in both hands caused him to have difficulty in moving his hands, which interfered with his daily life. The dermatologist referred him to our center of radiation oncology. When he visited the center of radiation oncology, his physical examination showed a generalized rash with widespread erosions and ulcers on both hands and both feet (Fig. 1). Biopsy showed atypical lymphoid cells infiltrating the epidermis and dermis, and the infiltrated cells were positive for CD3 and CD4 but negative for CD8 in the immunohistochemical staining results (Fig. 2).

**Figure 3.**
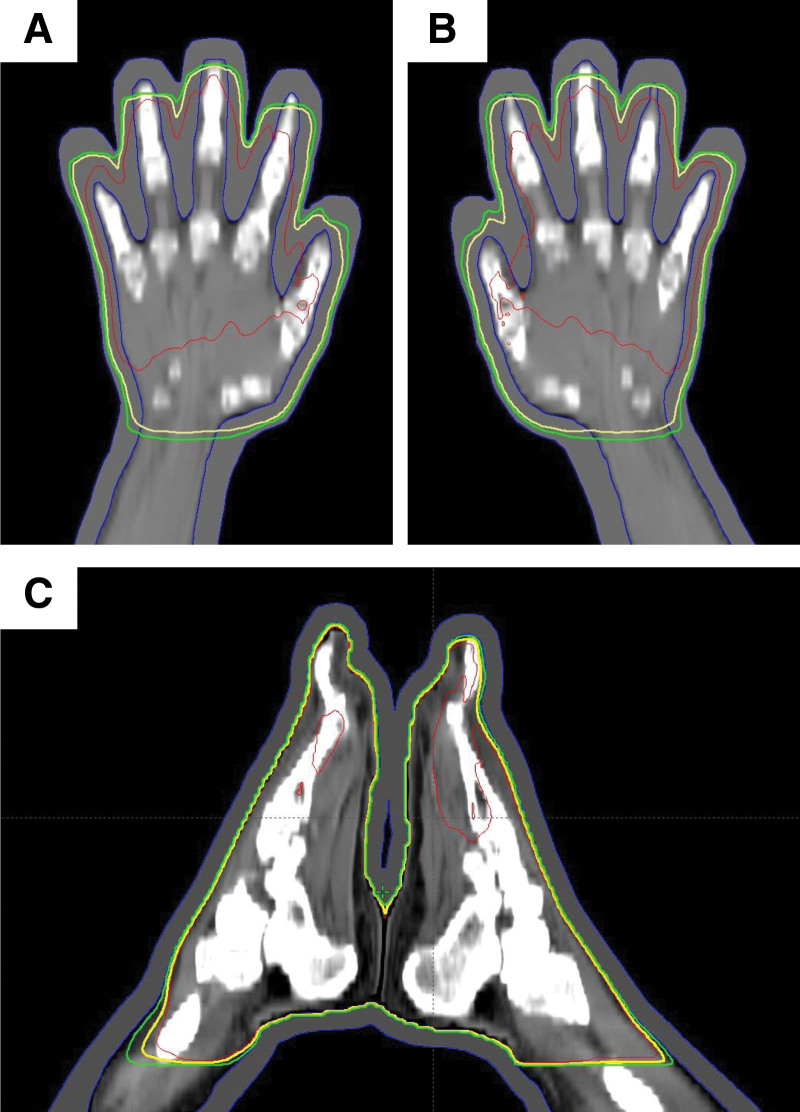
The images are the 3-dimensional conformal radiation treatment fields. the 1 cm-thick silicon bolus were placed on the skin as a tissue compensator. The photon beam could reach the entire lesions. (A) Left hand; (B) Right hand; (C) Both feet.

Thus, we restaged him as stage III CTCL and decided to treat using 3D-CRT to both his hands and feet with high dose for curative aim. To effectively include the interdigital skin area within the radiation field, we treated him using 3D-CRT with a 6 MV photon beam using the Truebeam linear accelerator (Varian, CA) instead of conventional electron therapy. The patient’s legs were placed in a frog leg position, and his hands and feet were fixed with a Vac-lok im-mobilization device. Additionally, to ensure the application of an adequate radiation dose to the skin tissue, a 1 cm-thick silicon bolus was placed on the hands and feet as a tissue compensator. A total dose of 50 Gy in 25 fractions was administered with a fractionated daily dose of 2 Gy 5 days/weeks for a total of 5 weeks (Fig. 3). We used parallel 6 MV photon beams to cover both the sole and plantar areas, and we did not include the patient’s fingernails to avoid the late side effect of dystrophic nails.

**Figure 4.**
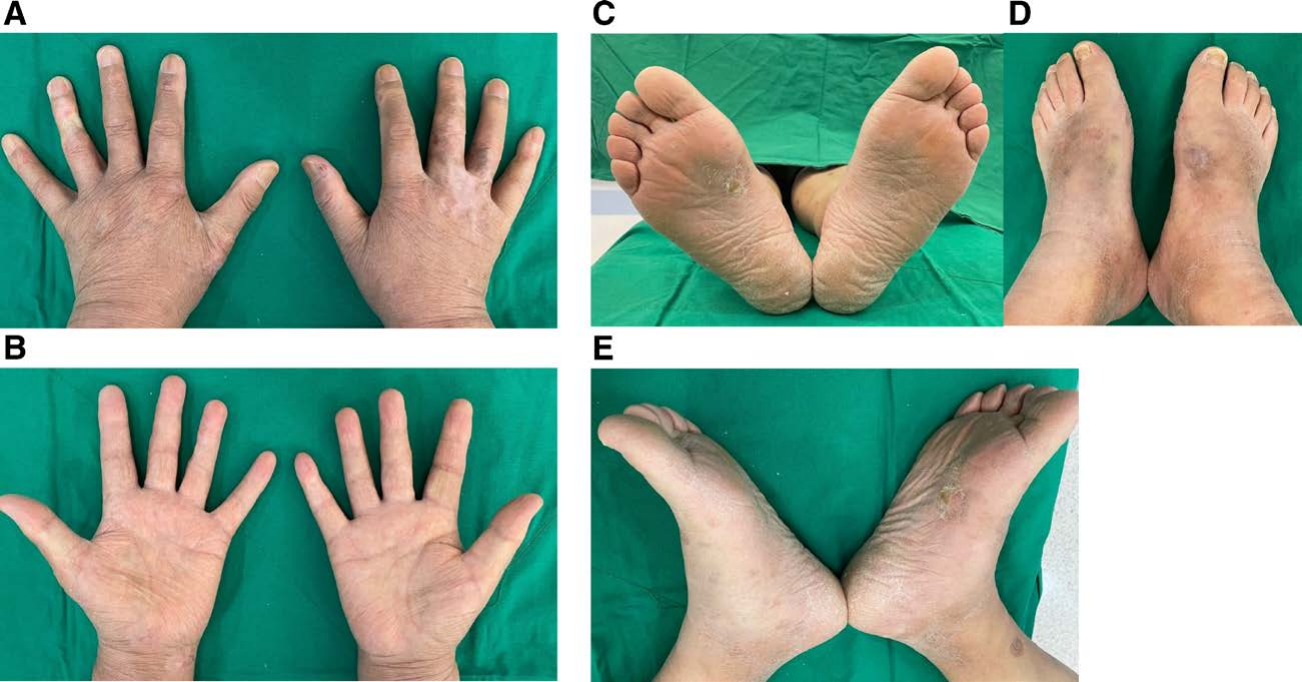
A follow-up picture taken 6 months after the end of radiotherapy shows complete resolution with only localized fibrosis on the irradiated skin. The patient continued the complete remission state for a total follow-up period of 24 months. Shown are the patient’s hands and feet. (A) The top of both hands; (B) the palm of both hands; (C) Both soles; (D) The top of both feet; (E) The inside of both feet.

At the 1-month follow-up after the end of radiotherapy, the irradiated skin had recovered to normal soft, smooth skin with localized fibrosis and hypopigmented skin, and the patient continued the complete remission state for a total follow-up period of 24 months (Fig. 4). There was no evidence of severe long-term side effects, such as skin necrosis and skin contraction. He reported an excellent quality of life, and his hands and feet could move freely. The written informed consent was obtained from the patient legal guardian/next of kin for the publication of his clinical data and accompanying images.

## 3. Discussion

CTCL is radiosensitive; thus, for patients with widespread disease, low-dose total skin electron beam therapy (TSEBT) is one of the most effective modalities for obtaining partial or complete responses for skin lesions in CTCL. For patients with localized and uni-lesional disease, radiation therapy may be considered in a limited number of the patients. The current National Comprehensive Cancer Network guidelines recommend a dose range of 12 to 36 Gy but also emphasize the advantages of using lower doses, including fewer complications and the ability to retreat progressive disease.^[5]^ The use of low-dose localized RT in the treatment of CTCL has also been studied, starting in 2009, with regimens of 2 × 4 Gy fractions^[6]^ and 1 × 7-8 Gy^[7]^ leading to complete response rates of 92% and 94%, respectively. However, Harrison’s group reported a response rate of 35% for patients with *T*3 disease who were given 10 to 20 Gy^[8]^ compared with 47% for patients with *T*3 disease who were given 30 to 40 Gy in a study by Navi et al^[9]^

The treatment for advanced-stage or refractory CTCL is largely palliative. Due to the rarity of the disease, there are few randomized controlled trials and large-scale prospective studies. Therefore, the treatment of advanced-stage CTCL remains challenging.^[10]^ However, in this case, we found that conformal local radiotherapy with a high radiation dose was effective and beneficial for maintaining quality of life. When the patient started radiotherapy in this study, we were concerned about severe toxicities, such as skin necrosis and skin contracture, due to the high dose. However, at 1 year after the end of radiotherapy, he showed only localized fibrosis and hypopigmentation on the irradiated skin. Thus, in those patients, radiation dose escalation with conformal radiotherapy is needed for curative purposes.

For successful local radiotherapy, the extent of radiation on the skin is important. Inclusion of the adjoining normal skin, up to 2 to 3 cm away from the margin of the visible lesions, is recommended, with a total dose of at least 40 Gy.^[11]^ With 3D-CRT, an adequate amount of therapeutic radiation can be delivered to lesions while significantly reducing the radiation to the surrounding normal tissues. 3D-CRT increases the effective treatment dose to malignant cells while avoiding the harmful effects on normal tissue.^[12]^

In conclusion, although the RT doses for patients with CTCL have been trending downward since past reports have shown adequate response rates and lower toxicity with RT doses of < 30 Gy, CTCL with advanced stage could require dose escalation with local radiotherapy for curative aim. Conformal radiotherapy with a high radiation dose could be effective and may have tolerable toxicities. Additionally, patients with advanced-stage CTCL require a multidisciplinary approach, as various combinations of skin-directed therapies, including radiotherapy, are curative.

## Author contributions

All authors have read and approved the final manuscript.

**Conceptualization:** Sun Young Lee.

**Data curation:** Sang Hee Youn.

**Supervision:** Sun Young Lee.

**Writing – original draft:** Sang Hee Youn.

**Writing – review and editing:** Sun Young Lee.

## References

[1] Hristov AC Tejasvi T Wilcox R. Cutaneous T-cell lymphomas: 2021 update on diagnosis, risk-stratification, and management. Am J Hematol. 2021;96:1313–28. 34297414 10.1002/ajh.26299 PMC8486344

[2] Kempf W Mitteldorf C. Cutaneous T-cell lymphomas-an update 2021. Hematol Oncol. 2021;39(Suppl 1):46–51. 34105822 10.1002/hon.2850

[3] Siegel RS Pandolfino T Guitart J. Primary cutaneous T-cell lymphoma: review and current concepts. J Clin Oncol. 2000;18:2908–25. 10920140 10.1200/JCO.2000.18.15.2908

[4] Lee SY Kwon HC Cho YS. The three dimensional conformal radiotherapy for hyperkeratotic plantar mycosis fungoides. Ann Dermatol. 2011;23(Suppl 1):S57–60. 22028574 10.5021/ad.2011.23.S1.S57 PMC3199424

[5] National Comprehensive Cancer Network. Primary Cutaneous Lymphoma (Version 2.2022). Available at: https://www.nccn.org/professionals/physician_gls/pdf/primary_cutaneous.pdf [access date June 8, 2022].

[6] Neelis KJ Schimmel EC Vermeer MH. Low-dose palliative radiotherapy for cutaneous B- and T-cell lymphomas. Int J Radiat Oncol Biol Phys. 2009;74:154–8. 18834672 10.1016/j.ijrobp.2008.06.1918

[7] Thomas TO Agrawal P Guitart J. Outcome of patients treated with a single-fraction dose of palliative radiation for cutaneous T-cell lymphoma. Int J Radiat Oncol Biol Phys. 2013;85:747–53. 22818412 10.1016/j.ijrobp.2012.05.034

[8] Harrison C Young J Navi D. Revisiting low-dose total skin electron beam therapy in mycosis fungoides. Int J Radiat Oncol Biol Phys. 2011;81:e651–7. 21489711 10.1016/j.ijrobp.2011.01.023

[9] Navi D Riaz N Levin YS. The Stanford university experience with conventional-dose, total skin electron-beam therapy in the treatment of generalized patch or plaque (*T*2) and tumor (*T*3) mycosis fungoides. Arch Dermatol. 2011;147:561–7. 21576575 10.1001/archdermatol.2011.98

[10] Gilson D Whittaker SJ Child FJ. British association of dermatologists and U.K. cutaneous lymphoma group guidelines for the management of primary cutaneous lymphomas 2018. Br J Dermatol. 2019;180:496–526. 30561020 10.1111/bjd.17240

[11] Eich HT Eich D Micke O. Long-term efficacy, curative potential, and prognostic factors of radiotherapy in primary cutaneous B-cell lymphoma. Int J Radiat Oncol Biol Phys. 2003;55:899–906. 12605967 10.1016/s0360-3016(02)04199-8

[12] Hansen JE Kim YH Hoppe RT. Primary cutaneous lymphomas. In: Halperin EC Perez CA Brady LW, (eds). Perez and Brady’s Principles and Practice of Radiation Oncology. Lippincott Williams & Wilkins 2019:1908–1920.

